# Relationship of ankle-brachial index, vibration perception threshold, and current perception threshold to glycemic variability in type 2 diabetes

**DOI:** 10.1097/MD.0000000000019374

**Published:** 2020-03-20

**Authors:** Chuangbiao Zhang, Meili Tang, Xiaohua Lu, Yan Zhou, Wane Zhao, Yu Liu, Yan Liu, Xiujie Guo

**Affiliations:** a Department of Endocrinology, First Affiliated Hospital of Jinan University; b College of Nursing, Jinan University, Guangzhou; c Department of Endocrinology, Qingyuan People's Hospital, Qingyuan, Guangdong Province, China.

**Keywords:** ankle-brachial index, continuous glucose monitoring, current perception threshold, glycemic variability, vibration perception threshold

## Abstract

To explore the relationship of glycemic variability with lower extremity arterial disease (LEAD) and diabetic peripheral neuropathy (DPN).

Seventy-eight patients with type 2 diabetes were enrolled. All patients underwent 72-hour dynamic blood glucose monitoring and obtained mean amplitude of glycemic excursions (MAGE), mean of daily differences (MODD), standard deviation of blood glucose (SD), largest amplitude of glycemic excursion (LAGE), mean blood glucose (MBG), T≥10.0 (percentage of time for blood glucose levels ≥10.0 mmol/L), T≤3.9 (percentage of time for blood glucose levels ≤3.9 mmol/L), and other glycemic variability parameters. In the meanwhile, in order to explore the correlation of glycemic variability parameters with ankle-brachial index (ABI), vibration perception threshold (VPT), and current perception threshold (CPT), all patients underwent quantitative diabetic foot screening, including ABI for quantitative assessment of lower extremity arterial lesions and VPT and CPT for quantitative assessment of peripheral neuropathy.

Patients were divided into abnormal CPT group (n = 21) and normal CPT group (n = 57) according to the CPT values. Compared with the normal CPT group, abnormal CPT group showed significantly higher levels of HbA_1c_, longer duration of diabetes, and higher levels of T≤3.9 (*P* < .05). However, there was no significant difference of MAGE, SD, LAGE, MODD, and other glycemic variability parameters between abnormal CPT group and normal CPT group (*P* > .05). Pearson correlation analysis or Spearman correlation analysis showed that ABI negatively correlated with MBG, T≥10.0, SD, LAGE, and MAGE (*P* < .05), but no correlation of ABI with T≤3.9 and MODD (*P* > .05) was shown. VPT showed a positive correlation with T≥10.0 (*P* < .05), but no correlation with other glycemic variability parameters (*P* > .05). There was no correlation between the other CPT values and the glycemic variability parameters (*P* > .05), except that the left and right 250 Hz CPT values were positively correlated with T≤3.9 (*P* > .05).

The higher the blood glucose levels, the severer the degree of LEAD and DPN lesions; the higher the incidence of hypoglycemia, the severer the degree of DPN lesions; the greater the fluctuation of blood glucose, the severer the degree of LEAD lesions. However, the glycemic variability was not significantly correlated with DPN.

## 1 Introduction

Glycemic variability is one of the most important parameters for evaluating blood glucose control. Hemoglobin A_1c_ (HbA_1c_) is able to reflect the average level of blood glucose within 3 months, but it is not able to reflect the variation of blood glucose fluctuations.^[1]^ Diabetic patients with similar HbA_1c_ control may have different risks of complications due to different extent of glycemic variability. Glycemic variability is even more harmful to chronic complications of diabetes than persistent hyperglycemia.^[2]^ Therefore, glycemic variability is intimately related to the onset and development of chronic vascular complications of diabetes:

1. Macrovascular complications: glycemic variability is significantly associated with the risk of cardiovascular disease such as severity of coronary heart disease and acute myocardial infarction in diabetic patients.^[3–5]^
2. Microvascular complications: glycemic variability y is associated with extent of albuminuria,^[6]^ and it is also a risk factor for diabetic retinopathy.^[7]^

Lower extremity arterial disease (LEAD) is one of the common chronic macrovascular complications of type 2 diabetes, and it is also an important predictor of non-healing foot ulcers and major amputation.^[8]^ However, diabetes with LEAD is one of the complications that is easily neglected by medical workers.^[9]^ Diabetic peripheral neuropathy (DPN) is often associated with LEAD, and it is a common microvascular complication with insidious onset and high disability.^[10]^ Although glucose variability is a very important parameter of predicting diabetic vascular complications, there are few studies on the relationship of glucose variability with LEAD and peripheral neuropathy.

Therefore, in this study, we aimed to explore the relationship between glycemic variability and ankle-brachial index (ABI), vibration perception threshold (VPT), and current perception threshold (CPT) to provide evidence for the relationship of glucose variability with diabetic LEAD and DPN. We assessed the extent of glucose variability using continuous glucose monitoring (CGM) system and quantitatively assessed the extent of LEAD by measuring ABI. And we used VPT and CPT to quantitatively assessed the extent of DPN.

## 2 Methods

### 2.1 Study design and subjects

This cross-sectional observational study was conducted from April 2018 to June 2019 in the Department of Endocrinology, the First Affiliated Hospital of Jinan University. Patients with type 2 diabetes who underwent LEAD and DPN screening, as well as were monitored for dynamic blood glucose by CGM, were enrolled in this study. The inclusion criteria were as follows:

1. meeting the Diabetes Diagnostic Criteria of the American Diabetes Association in 2011
2. 25 to 75 years old
3. receiving a stable hypoglycemic therapy based on glucose control goals under the guidance of professional physicians.

Exclusion criteria include:

1. type 1 diabetes or other special types of diabetes
2. acute complications of diabetes, namely diabetic ketoacidosis and hyperosmolar hyperglycemic state, etc
3. using neurotoxic drugs, such as chemotherapy drugs, etc
4. folic acid and vitamin B12 deficiency, as well as heavy metal poisoning
5. severe mental behavior disorder or mental retardation, non-cooperating with examination and treatment
6. hyperthyroidism or hypothyroidism
7. history of malignant tumors
8. severe heart, liver, brain, kidney, and other important organs related diseases
9. connective tissue diseases
10. severe cervical and lumbar lesions (neural root compression, spinal canal stenosis, etc)
11. Guillain–Barré syndrome
12. lower extremity venous embolism and lymphangitis

This study strictly adheres to the principles of the Helsinki Declaration and each participant provided written informed consent. The research protocol was reviewed and approved by the Ethics Committee of the First Affiliated Hospital of Jinan University. A total of 78 patients with type 2 diabetes were enrolled in the study.

### 2.2 Demographic and clinical data

The demographic and clinical information of all participants enrolled were collected and organized by professionally trained observers, including: age, gender, duration of diabetes, smoking history, height, weight, BMI, systolic blood pressure, diastolic blood pressure, etc. Blood pressure was measured 3 times after the participants had rested for at least 30 minutes, and the average of 3 records was calculated for further analysis.

### 2.3 Laboratory data

All participants fasted as requested for more than 8 hours. Fasting venous blood and urine samples were collected at 7 am the next morning to test the clinical and biochemical parameters. Detection of serum uric acid, total cholesterol, triglyceride, low density lipoprotein-cholesterol, and high-density lipoprotein cholesterol was done using a fully automated biochemical analyzer (Model 7600 Series, Hitachi, Tokyo, Japan). The HbA_1c_ levels were measured by high-performance liquid chromatography (D-10 kit, Bio-Rad, USA).

### 2.4 CGM and glycemic variability parameters

The dynamic blood glucose levels of patients were monitored using a dynamic blood glucose monitoring system (MiniMed Paradigm 722, Medtronic MiniMed, USA). The dynamic blood glucose monitoring probe was implanted into the subcutaneous tissue of the abdomen of the subject on the first day; then it was removed from the body on the fourth day. Patients were continuously monitored by the system for 72 hours, and the values of probe measurements were automatically stored in the system every 5 minutes. During the monitoring period, the fingertip blood glucose levels were measured at least 4 times daily and matched with the probe measurement values at the same time. When the dynamic blood glucose monitoring system failed, professional clinical staffs helped to solve the problem. The data was extracted using the analysis software (CareLink, Medtronic MiniMed, USA) in the dynamic blood glucose monitoring system to evaluate the accuracy of the data. The best accuracy evaluation standard^[11]^ was:

1. Daily pair of matching probe measurement values and fingertip blood glucose values for each subject were ≥3; the correlation coefficient between the probe measured values and the fingertip blood glucose levels was ≥0.79
2. When the difference between the highest fingertip blood glucose levels and the lowest fingertip blood glucose levels was ≥5.6 mmol/L, the mean absolute difference (MAD) was ≤28%; when the difference between the highest and the lowest blood glucose levels of fingertips was <5.6 mmol/L, then the MAD was ≤18%.

Glycemic variability parameters were analyzed based on blood glucose curve data monitored on day 2 and day 3. Glycemic variability parameters included mean amplitude of glycemic excursions (MAGE), mean of daily differences (MODD), standard deviation of blood glucose (SD), maximum glycemic variability, largest amplitude of glycemic excursion (LAGE), mean blood glucose (MBG), percentage of time for blood glucose ≥10.0 mmol/L (T≥10.0), and percentage of time for blood glucose ≤3.9 mmol/L (T≤3.9).^[12]^ During the 24-hour monitoring period, the glycemic variability of the subject that was greater than 1 SD (the SD of the measured value during the 24-hour period of the subject) was considered as effective fluctuation, and MAGE was the average of all effective fluctuations to assess intraday blood glucose fluctuations.^[13]^ MODD was the average of the absolute values of the difference between the measured value and the corresponding measured value during the 2 consecutive 24-hour monitoring periods, which was an indicator of the average daily glycemic variability in response time.^[14]^ The difference between the maximum and minimum blood glucose values during the measurement period was LAGE, which was used to evaluate the magnitude of the maximum glycemic variability. The SD was used to evaluate the dispersion of the average blood glucose levels of the subject throughout the day. MBG was the average of all blood glucose values on a dynamic blood glucose monitoring system.^[15]^ T≥10.0 represented the percentage of time taken for blood glucose levels ≥10.0 mmol/L during the monitoring period. Subjects were considered by the system to be the onset of hypoglycemic events when the blood glucose level was below 3.9 mmol/T, and T≤3.9 represented the percentage of time when blood glucose ≤ 3.9 mmol/L during the monitoring period to evaluate the incidence of hypoglycemia.

### 2.5 ABI test and decision criteria

After the completion of dynamic blood glucose monitoring, bilateral ABIs were detected under the same conditions using a peripheral vascular diagnostic system (Vista AVS, Summit Doppler Systems, Inc. USA). All patients were tested by trained professional staffs using the same detection method, which was as follows: the subject was asked to rest in a supine position, and the limbs were naturally laid flat so that the arms, ankles, and feet were fully exposed. After the cuff and the pressurized tube were connected together, they were tied up them to the arms and ankles of the subject, respectively. When measuring the arterial pressure, the lower edge of the arm band should be placed 2 to 3 horizontal fingers above the elbow fossa. When measuring the ankle artery pressure, the lower edge of the arm band was placed at 2 to 3 horizontal fingers above the medial mallus to detect the bilateral systolic blood pressure of the brachial artery and the systolic pressure of the ankle artery (including the systolic pressure of the dorsalis pedis artery and the systolic pressure of the posterior tibial artery) of the subject. The higher value of the systolic pressure of the dorsalis pedis artery or the posterior tibial artery was considered as the systolic pressure of the ankle artery to calculate the ABI. ABI referred to the ratio of systolic pressure of the ankle artery to the systolic pressure of the ipsilateral radial artery. In the ABI on both sides, the lower value of 1 side was taken as the ABI value of the patient. The normal reference value of ABI was defined as 0.91 to 1.30. LEAD could be diagnosed when ABI≤0.90, and ABI ranging from 0.71 to 0.90 was considered as mild arterial disease, ABI ranging from 0.41 to 0.70 was for moderate arterial disease, and ABI≤0.40 was for severe arterial disease.^[16]^ Therefore, the lower the ABI value, the severer the arterial lesions.

### 2.6 VPT detection and decision criteria

At the end of the dynamic blood glucose monitoring, the vibration sensitivity threshold of the patient's feet was detected under the same conditions using a digital vibration sensation quantitative tester (Sensiometer A200, Laxons Technology Co. Ltd, Beijing, China). All patients were tested by trained professional staffs using the same detection method as follows: the professional staffs explained and demonstrated the VPT detection process to the patient before the formal examination asking the patient to relax, lie on his back, and close his eyes. The vibrating head of the inspector was vertically contacted with the patient's examination site, and the knob was started from 0 and gradually increased to a speed of 1 V/s. The voltage values were recorded when patients started to feel the vibration. The examination site was the back of the first toe. The test on each side was repeated 3 times and the average value was taken as the value of that side. Both sides of the foot were tested for the same VPT, and the value of the side with larger vibration threshold value was taken as the VPT value of the patient. VPT <15 V was considered as normal feeling, 16 to 24 V indicated mild sensory abnormality, and VPT greater than 25 V was considered as severe sensation abnormality.^[17]^ Therefore, the larger the VPT value, the severer the neuropathy.

### 2.7 CPT detection and decision criteria

After completing the dynamic blood glucose monitoring, the Neurometer (Neurometer CPT/C, Neurotron Inc., Baltimore, USA) was used to detect the current sensory threshold of patients’ feet under the same conditions. All patients were tested by trained professionals using the same operation method as follows: patient was requested to be completely relaxed, and the inside/outside of the first toe of the 2 feet was selected as a test point to reflect the superficial nerve and deep nerve function; after turning on the button, the sinusoidal current stimulus was slowly increased from 0.00 to 9.99 mA to measure CPT with 3 predetermined frequencies (2000 Hz, 250 Hz, 5 Hz). Until the patient reported the sensation, the stimulus was turned off. Then the intensity level was lowered and the re-measurement was performed. This process was cycled until a minimum and constant threshold was determined, which was the sense current threshold CPT value. The corresponding CPT values of the 2 feet at 2000 Hz, 250 Hz, and 5 Hz were measured. The normal range of CPT was 179 to 523 mA × 100 for 2000 Hz, 44 to 208 mA × 100 for 250 Hz, and 18 to 170 mA × 100 for 5 Hz. If the CPT value was in the normal range, it was considered an normal CPT. Hypersensitivity was considered for CPT values below normal range at any frequency of both feet. A CPT value higher than the normal value at any frequency of the 2 feet was considered to be hypoesthesia. CPT value at any frequency of the bipedal was hypersensitivity or (and) hypoesthesia should be considered as paresthesia, that is, abnormal CPT. Therefore, if the CPT value was too high or too low, it should be an abnormal CPT.

### 2.8 Statistical analysis

Continuous variable data was expressed as mean ± SD if the data was consistent with the normal distribution. The data was expressed as median (interquartile range) if the data was skewed distribution. Categorical data was expressed as frequency (percentage). Normal distribution data, skewed distribution data, and categorical data were compared using Student *t* test, Mann–Whitney *U* test, and Chi-Squared test, respectively, when comparing differences between the 2 groups. Correlation analysis between variables was made using Pearson correlation analysis (data that accords with normal distribution) or Spearman correlation analysis (data that does not accord with normal distribution). All statistical analyses were performed using SPSS 19.0 software. *P* < .05 was considered statistically significant.

## 3 Results

### 3.1 Comparison of clinical characteristics between abnormal CPT group and normal CPT group

The overall clinical characteristics of the 78 patients with type 2 diabetes and that of the subgroups (abnormal CPT group and normal CPT group) are shown in Table 1. Compared with the normal CPT group, the abnormal CPT group showed significantly higher HbA_1c_ levels and longer duration of diabetes (*P* < .05), but there was no significant difference of other parameters such as age, gender, systolic blood pressure, diastolic blood pressure, smoking, uric acid, and blood lipids between abnormal CPT group and normal CPT group (*P* < .05).

**Table 1:**
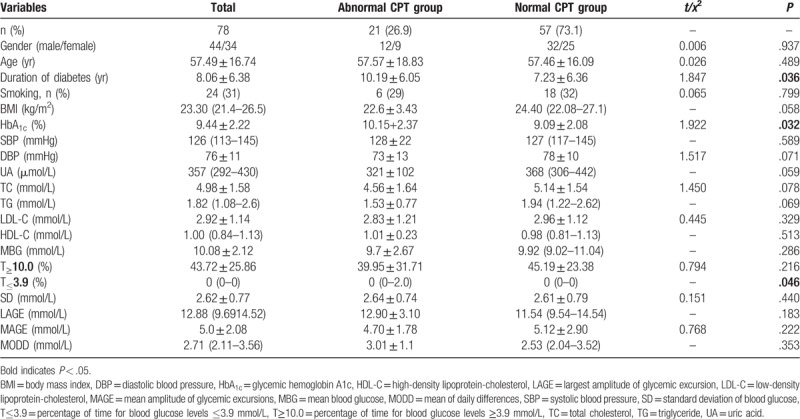
Comparison of clinical characteristics between abnormal CPT group and normal CPT group.

### 3.2 Correlation analysis of glycemic variability parameters with ABI, CPT, and VPT

The correlation analysis of glycemic variability parameters with ABI, CPT, and VPT values is summarized in Table 2. Pearson correlation analysis or Spearman correlation analysis demonstrated that ABI showed a negative correlation with MBG, T≥10.0, SD, LAGE, and MAGE (*P* < .05), but no correlation with T≤3.9 and MODD (*P* > .05) was shown; VPT showed a positive correlation with T≥10.0 (*P* < .05), but no correlation with other glycemic variability parameters (*P* > .05). There was no correlation between the other CPT values and the glycemic variability parameters (*P* > .05), except that the left and right 250 Hz CPT values were positively correlated with T≤3.9 (*P* > .05).

**Table 2:**
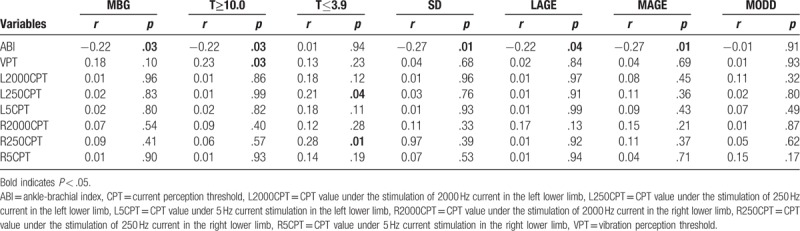
Correlation analysis of glycemic variability parameters with ABI, CPT, and VPT.

## 4 Discussion

Two large prospective clinical studies, the Diabetes Control and Complications Trial^[18]^ and United Kingdom Prospective Diabetes Study^[19]^ have demonstrated that intensive blood glucose control in early type 2 diabetic patients reduced the risk of diabetic vascular complications and have established HbA_1c_ as the “gold standard” for blood glucose control. However, the Action to Control Cardiovascular Risk in Diabetes Study Group^[20]^ showed that intensive glucose lowering in patients with type 2 diabetes who already have cardiovascular diseases or cardiovascular risk factors significantly increased mortality. And subsequent Veterans Affairs Diabetes Trial^[21]^ showed that intensive glucose control had no significant effect on cardiovascular events, death, and microvascular complications. These findings suggest that the occurrence of diabetic complications is not only related to elevated blood glucose levels, and HbA_1c_ as the gold standard is not the only indicator for predicting diabetic complications. HbA_1c_ is not able to detect severe hyperglycemia, hypoglycemia, and glycemic variability in time. There are certain limitations to HbA_1c_ as the sole target for diabetes control. Therefore, dysglycemia does not only refer to elevated blood glucose, but also includes hypoglycemia and glycemic variability, which is closely related to chronic complications of diabetes. Studies have shown that^[22]^ the risk of macrovascular complications in patients with type 2 diabetes is determined by the combination of fasting blood glucose, postprandial blood glucose, HbA_1c_, hypoglycemia events, and glycemic variability. Similarly, persistent hyperglycemia, hypoglycemia, and glycemic variability may also be risk factors for DPN.^[23]^

LEAD is one of the common chronic macrovascular complications of type 2 diabetes and one of the main causes of foot ulcers, disability, and death in diabetic patients. ABI is a commonly used indicator for assessing the degree of arterial ischemia in the lower extremities, and it is commonly used for LEAD screening for its advantages of low cost, simplicity, high reproducibility, and specificity.^[17]^ In this study, we examined the extent of LEAD by detecting ABI, and we explored the relationship between ABI and glycemic variability parameters. It showed that ABI had a significant negative correlation with MBG and T≥10.0. Therefore, the greater the increase in blood glucose, the severer the LEAD, which is inconsistent with the findings of individual studies.^[24]^ This inconsistency may be related to the relatively long course and the relative high blood glucose levels of the patients enrolled in this study. In the United Kingdom Prospective Diabetes Study, a 10-year follow-up visit demonstrated that lowering blood glucose reduced the incidence of macrovascular complications.^[25]^ MAGE, SD, LAGE, and MODD are commonly used parameters to evaluate glycemic variability, the most representative of which is MAGE.^[26]^ In this study, ABI showed a significant negative correlation with MAGE, SD, and LAGE, suggesting that glycemic variability was related to the degree of LEAD. The greater the fluctuation of blood glucose, the heavier the LEAD. The possible mechanism of the above findings is as follows: vascular endothelial dysfunction is considered to be the basis of macrovascular complications of type 2 diabetes, and hyperglycemia may cause vascular endothelial cell dysfunction, which may accelerate the occurrence of diabetic macrovascular complications.^[27]^ In the meanwhile, glycemic variability aggravated oxidative stress in patients with type 2 diabetes and further damaged endothelial cells, leading to the macrovascular complications of diabetes.^[28]^ However, in this study, ABI was not correlated with T≤3.9, suggesting that hypoglycemia was not associated with the extent of LEAD, which was inconsistent with some studies, including Diabetes Control and Complications Trial study.^[24,29]^ This inconsistency was possibly related to the relatively high blood glucose levels of the patients enrolled in this study, all of them were hospitalized patients, the regulation of blood glucose levels during hospitalization by resident physician, and the relatively low risk of hypoglycemia.

DPN is one of the most common chronic complications of diabetes. It is a major risk factor for diabetic foot ulcers even leading to amputation which seriously affects the survival and quality of life of diabetic patients.^[30,31]^ Therefore, early screening of DPN is particularly important. Quantitative sensory testing (QST) is a kind of neurophysiological test that can quantitatively analyze the degree of neuropathy by stimulating skin receptors using vibration, current, cold, and heat. Traditional QST includes VPT and temperature threshold, while modern QST includes CPT. Both the American Peripheral Neuropathy Association and the American Diabetes Association recommended using QST as a diagnostic criterion for the diagnosis of DPN.^[32]^ The VPT^[33]^ and CPT^[34]^ used in this study were reliable quantitative screening methods for early or even asymptomatic DPN. In this study, the abnormal CPT group showed higher HbA_1c_ levels and longer duration of diabetes than the normal CPT group, and the VPT value was positively correlated with T≥10.0, which was consistent with many large studies,^[19,35]^ suggesting that with an increase in blood glucose and prolonged course of diabetes, the development of diabetic microvascular complications can be promoted. Although many studies^[24,36]^ have shown that hypoglycemia increased the risk of atherosclerotic macrovascular complications, the relationship between hypoglycemia and DPN is still not clear.^[23]^ This study showed that T≤3.9 was higher in the abnormal CPT group than in the normal CPT group, and the 250 Hz CPT value was positively correlated with T≤3.9, suggesting that the higher the incidence of hypoglycemia, the severer the peripheral neuropathy, which may be associated with hypoglycemia.^[37,38]^ All these above may be related to the important aspects of the pathogenesis of DPN^[37,38]^ including^[39]^ oxidative stress and inflammatory factor release, etc. In this study, it is worth noting that the VPT values and CPT values of each frequency were not correlated with the important glycemic variability parameters, which were SD, LAGE, MAGE, and MODD. And there was no difference of these above glycemic variability parameters between the abnormal CPT group and the normal CPT group. However, a study^[40]^ found that patients with type 2 diabetes with well-controlled HbA_1c_ (HbA_1c_ < 7%) in the DPN group showed higher levels of SD and MAGE than the non-DPN group. The average of HbA_1c_ levels of the patients enrolled in our study was 9.44%, which was higher than the above studies, and it might explain the difference in results to some extent. It may be inferred that in patients with good HbA_1c_ control, glycemic variability was closely related to DPN, but this kind of relationship was unclear in patients with poor HbA_1c_ control. However, another recent study^[41]^ that enrolled patients with poor HbA_1c_ control (mean HbA_1c_ was 9.87%) found higher levels of SD, MAGE, and MODD in DPN group than the non-DPN group. However, in their study, the diagnostic criteria for DPN included symptomatic, physical signs, and nerve conduction studies abnormalities. While in our study, some of our patients showed abnormal QST but no DPN symptoms, and QST may be able to detect early or even asymptomatic DPN patients earlier than nerve conduction studies.^[42]^ Therefore, the DPN patients in our study may be milder or earlier than that in their study, which may cause inconsistencies to some extent. Whether the effects of glycemic variability differ in the early or late stages of DPN remains to be confirmed by further studies. A recent study^[43]^ that enrolled patients with characteristics similar to ours (mean HbA_1c_ was 8.7%) also used the tuning fork vibration sense to quantitatively evaluate DPN, showing that SD and MAGE were not related to DPN, which was consistent with our findings.

Our study still has some limitations. First, in this study, cross-sectional design was used, and causal relationship cannot be identified. Second, our sample size was relatively small and we did not follow up these patients, so we could not establish the prognostic role of CPT in diabetes. Third, other factors like drugs that could affect glycemic variability were not taken into consideration.

## 5 Conclusion

In summary, we found that the higher the blood glucose levels, the severer the LEAD and DPN lesions; the higher the incidence of hypoglycemia, the severer the degree of DPN lesions; the greater the glycemic variability, the severer the LEAD lesions. However, there was no significant correlation between DPN and glycemic variability. Therefore, in order to delay the development of LEAD and DPN, clinicians should not only focus on the blood glucose levels and the HbA_1c_ standard when it comes to the blood glucose management, but also reduce the glycemic variability and the incidence of hypoglycemia to smoothly lower the blood glucose level of diabetic patients.

## Author contributions

Acquisition of data: Meili Tang, Wane Zhao, Yu Liu, Yan Liu, Xiujie Guo; analysis and interpretation of data: Chuangbiao Zhang, Meili Tang, Xiaohua Lu; conception and design of the research: Chuangbiao Zhang, Xiaohua Lu, Yan Zhou; drafting the manuscript: Chuangbiao Zhang, Meili Tang; revision of manuscript for important intellectual content: Xiaohua Lu, Yan Zhou; statistical analysis: Chuangbiao Zhang, Xiaohua Lu. All authors read and approved the final manuscript.
